# 

**Published:** 1904-04-09

**Authors:** 


### NOTICE TO CORRESPONDENTS.

All MSS., Letters, Books for Review, and other matters intended for the Editor should be addressed The Editor, "The Hospital," The Hospital Buildings, 28 & 29 Southampton Street, Strand, W.C.

The Editor cannot undertake to return rejected MS., even when accompanied by stamped directed envelope.

**Notice to Advertisers and Subscribers.**

All Advertisements, Orders for Copies of The Hospital, and Business Communications should be addressed to **THE MANAGER (Not to the Editor),** The Hospital, 28 & 29 Southampton Street, Strand, London, W.C.

The Cost of Subscription, post free, is as follows:—Per week, 3½d.; per quarter, 3s. 9d.; per half-year, 7s. 6d.; per annum, 15s. Foreign and Colonial:—Per annum, 19s. 6d., payable, in all cases, in advance.

**NOTICE.—Vol. XXXIV. of "The Hospital" (April 1903 to September 1903), in handsome cloth binding, is now on sale at the office of this Journal, price 10s. 6d. Cases for binding the Numbers contained in this Volume 2s. Index to Vol. XXXIV., 2½d., post free.**

**The Monthly part for April is now ready. Price 1s., by post, 1s. 3d.**

### The Student of Medicine.

### APPOINTMENTS.

**Charles N. Burton**, L.R.C.P.Lond., M.R.C.S.Eng., Anæsthetist to the Gordon Hospital, Vauxhall Bridge Road. **M. R. Cleary**, F.R.C.S., L.R.C.P.Irel., Certifying Factory Surgeon for the Hospital District, co. Limerick. **W. Frank Colclough**, M.D., B.C.Cantab., Medical Officer to Out-Patients, Yeatman Hospital, Sherborne. **A. H. Cook**, M.B., M.R.C.S., L.R.C.P., Honorary Medical Officer to the Infants' Hospital, Hampstead. **T. H. Goodman**, M.R.C.S.Eng., L.S.A., Certifying Factory Surgeon for the Haverhill District, Suffolk. **Stuart Hackworth**, M.D.Lond., D.P.H.Camb., M.R.C.S., L.R.C.P., Medical Adviser to the Hanley Education Committee. **T. N. Kelynack**, M.D., M.R.C.P., Honorary Physician to the Infants' Hospital, Hampstead. **J. H. Laidlaw**, M.D., Clinical Assistant to the Chelsea Hospital for Women. **Frederic W. Longhurst**, L.R.C.P.Lond., M.R.C.S.Eng., Anæsthetist to the Gordon Hospital, Vauxhall Bridge Road. **S. Moritz**, M.D.Wurzburg, M.R.C.P.Lond., Lecturer on Diseases of the Throat and Nose at the Victoria University. **John Austin O'Dowd**, M.B.Lond., M.R.C.S., District Medical Officer and Public Vaccinator of the Dudley Union. **W. R. Pirie**, M.B., Ch.B., D.P.H.Aberd., House Surgeon to the Royal Aberdeen Hospital for Sick Children. **Percy C. Rayner**, M.B., Ch.B.Edin., Medical Officer and Public Vaccinator for the Brailsford District of the Ashbourne Union. **Eva A. Robertson**, M.B., Ch.B.Edin., Resident Medical Officer for the Craiglockhart Poorhouse. **W. H. Maxwell Telling**, M.D., B.S.Lond., M.R.C.P., Assistant Physician to the Hospital for Women and Children, Leeds. **J. Tennant**, M.B., C.M.Edin., Medical Officer of Health for the Brumby and Frodingham Urban District.

IN THE PRESS. READY SHORTLY.

### A COMPLETE HANDBOOK OF MIDWIFERY.

By J. K. WATSON, M.D., M.B., C.M.

Author of "A Handbook for Nurses," "Examination of the Urine," &c., &c.

This handbook has been designed to meet the requirements of the Central Midwives Board, and is an up-to-date text-book of **all** that the midwife requires to know of the subject treated. The work is produced with the object of rendering it unnecessary for midwives, during their preparation for examination, to consult the larger and more expensive works on the subject written specially for medical men.

**The illustrations are very complete, numbering some 143 separate diagrams, most of which have been specially prepared for this book.**

Crown 8vo., about 350 pp., bound in cloth boards, **6/-** net, **6/4** post free.

NOW READY.

### THE MIDWIVES' POCKET BOOK.

By HONNOR MORTEN.

With Appendix containing particulars and rules of the Central Midwives Board.

Bound in cloth, 1/- post free.

| | | |
|---|---|---|
| 2/- Post Free. | **PRACTICAL GUIDE TO SURGICAL BANDAGING AND DRESSINGS.** By Wm. Johnson Smith, F.R.C.S. Demy 16mo. profusely illustrated (suitable for Apron pocket), cloth - - - - - - - | 2/- Post Free. |
| 1/- net, 1/1 Post Free. | **THE EXAMINATION OF THE URINE.** By J. K. Watson, M.D., M.B., C.M., Author of "A Handbook for Nurses." Demy 16mo. cloth boards - - - - - - | 1/- net, 1/1 Post Free. |

*Of all Booksellers, or of*

THE SCIENTIFIC PRESS, LTD., 28 & 29 Southampton Street, Strand, London, W.C.

*Complete Catalogue of Nursing Handbooks post free on application.*

